# Clinical Immunology of Cholera - Current Trends and Directions for Future Advancement

**DOI:** 10.6026/97320630013352

**Published:** 2017-10-31

**Authors:** Francesco Chiappelli, Allen Khakshooy, Nicole Balenton

**Affiliations:** 1 Laboratory of Human Psychoneuroendocrine-Osteoimmunology; School of Dentistry, UCLA Center for the Health Sciences, Los Angeles, CA 90095-1668; 2 Evidence-Based Decision Practice-Based Research Network, DGSO, Los Angeles, CA 91403; 3 Department of the Health Sciences, CSUN, Northridge, CA 91330; 4 Rappaport Faculty of Medicine, Technion-Israel Institute of Technology, Haifa, Israel 3109601

**Keywords:** vibrio Cholera (vCh), innate immunity, antigen-dependent immunity, cell-mediated immunity, humoral immunity

## Abstract

Cholera remains a feared, aggressive, infectious and lethal disease today, despite several decades of intense research, concerted public health modalities designed to prevent, and to control outbreaks, availability of efficacious vaccines aimed at containing its contagious spread, and effective patient-centered medical interventions for reducing morbidity and mortality. Despite these advances, cholera still strikes communities around the world, especially in countries and regions of the globe where medical and nursing care cannot be as effectively proffered to the population at risk as in First World economies. Case in point, the number of suspected cholera cases that currently afflicts Yemen escalates at an "unprecedented rate", according to the World Health Organization. Here, following a brief introduction of the history of the medical knowledge about cholera, we discuss current trends of our understanding of clinical immune surveillance against the bacillus that causes cholera, vibrio Cholera (vCh). We cite the current state of best available evidence about anticholera vaccines, and outline certain directions for future study to characterize the clinical immunology of cholera.

## Background

Advances in our understanding of the physiopathology and clinical management of Cholera have been remarkable over the last two centuries. The early theory of miasma, a term that can be loosely translated from its Greek root as 'polluted' or 'bad' air, which had been shared by many cultures over several centuries, was integrated into Western medical practice in large part through the work of Sebastian Petrycy in Krakow in the early decades of the 1600's. The causative factor of 'bad air' was well engrained as a putative etiology factor for the cholera outbreaks in Paris and London in the first half of the 19th century.

John Snow, a British medical doctor and epidemiologist shook proposed that cholera was not due to bad air, but to bad water. He applied novel mathematical concepts of estimation - which today we would describe as biostatistics - to accurately pin point the focal point of origin of the London cholera outbreak to one specific water pump in the suburb of Soho along the Thames. Snow followed the lead of the Belgian-German mathematician, Peter Gustav Lejeune Dirichlet, who had formally defined the Fourier-derived analytical function that characterized progression and convergence of a series of event from a focal point. In 1854-6, Snow disseminated his findings in national medical meetings across the British Isles and international meetings across Europe, vigorously defending that cholera could be contained only if and when the drinking water was sanitized. The medical establishment remained entrenched in the miasma theory for the etiology agent of cholera, and his work was largely ignored.

Filippo Pacini, a medical doctor in the Grand Duchy of Tuscany and professor at the University of Pisa and subsequently Florence, tested Snow's proposition by seeking to elucidate what might be causing 'bad' drinking water during the violent outbreak of cholera that ravaged Tuscany in the 1850's. Pacini, well trained in Fracastoro's germ theory of contagion, postulated an identifiable germ in the water, the putative etiological factor for cholera. He compared watery stool samples from cholera victims to stools from patients deceased from ailments other than cholera. He described a bent rod-like germ, vibrio Cholera (vCh), in all cholera victims, which was present cholera-inducing drinking water as well, in his 1854 original publication. He followed this work with several additional treatises on the subject in the decade that followed until his last publication on the subject in 1880. His work was mostly written in Italian, which had ceased to be the language of science several decades earlier; and it was largely ignored. Pacini died deprived of the recognition of having identified vCh as the water-borne causative and contagious factor for cholera.

Building on the early work of Louis Pasteur, the German medical doctor, Robert Koch, established himself at the forefront of the emerging field of bacteriology in the late 1800's. He developed four postulates, sine qua non's to establish the virulence of an infectious agent: a) the microorganism must be found in abundance in all organisms suffering from the disease, but should not be found in healthy organisms; b) the microorganism must be isolated from a diseased organism and grown in pure culture; c) the cultured microorganism must be capable of causing the disease when introduced into a healthy organism; and d) the microorganism must be isolated anew from the inoculated, diseased experimental host and confirmed as being identical to the original specific causative agent. By these criteria, he discovered and characterized the causative agent for anthrax (1876), tuberculosis (1882), cholera (1883/4), and several others. Koch's work was widely embraced.

Along parallel lines, British physician, Edward Jenner, developed the first successful vaccination protocol against Variolae vaccinae (smallpox of the cow) in 1798, laying the foundations for humoral immunity. It was not until Russian biologist Ilya Ilyich Mechnikov that the basic biology of myeloid cell-mediated immunity was first elucidated. He described the process by which foreign bodies, such as vCh bacillus, are phagocytosed and processed into individual "nonself" antigens. Several decades later, Macfarlane Burnet, inspired by Niels Jerne, proposed that the major histocompatibility complex (MHC) presented by myeloid cells (i.e., antigen-presenting cells, APC) is recognized by T lymphoid cells as "self". When APC present "self+nonself" and triggers a cell-mediated immune response. Thus, "self+nonself" presented by MHC class II trigger T cells (CD3+) that express the CD4 membrane glycoprotein cluster of differentiation (CD4+), whereas "self+nonself" presented by MHC class I trigger CD8+ T cells.

### Clinical Immunology of vCh: Current Trends

Clinical immune surveillance is a complex and finely regulated process that involves two primary dimensions [1, 2, 3, 4] innate immunity, which includes the phagocytosis and processing of bacteria, such as the diverse sero-groups of vCh, among which O1 and the less virulent O139 strain, mainly by myeloid APC's. Upon antigen presentation, APC's become activated and can transit from an M0 to M1 or M2 differentiation state. It is not clear to this date if vCh induces M1 or M2 specialization. During APC activation, inflammatory markers of innate immunity are produced, including interleukin [IL]-1b, and tumor necrosis factor [TNF]-a), which aid in the engagement of CD3+ lymphoid T-cell activation. It is both remarkable and alarming that, compared to other infectious diseases, we have relatively little knowledge about the timeline of the humoral, cellular, and molecular characteristics of the innate immune responses to vCh- O1, vCh-O139, or any other vCh serogroup.

CD3+ lymphoid cell activation follows, is dependent upon "self+nonself" APC presentation, and signifies the engagement of antigen-dependent acquired immunity. It commences with the expression of the chain of the IL-2 receptor, CD25, and other cellular markers of activation (e.g., transferrin receptor, CD71). Activated CD3+ cells produce humoral immunity factors, such as IL-2, the T cell growth factor, which act in concert to sustain and expand antigen-dependent clinical immune surveillance. T cell immunology is a complex umbrella of response, which involves the maturation of CD3+ cell from a naive state (CD45RA+) to a memory state - immune memory to the presented "nonself" antigen - CD45RA-CD45R0+. Both naïve and memory CD3+CD4+ and CD3+CD8+ cells can be resting (CD25-) or activated (CD25+).

Bacteria, such as vCh, engage naive CD4+ T cells (CD3+CD4+CD45RA+), activate them into CD3+CD4+CD25+CD45RA-, and lead to the generation of CD3+CD4+CD25+/-CD45R0+ memory cells. From a humoral immunity perspective, this process of CD4+ cell maturation coincides with a shift in the profile of T cell secreted cytokines from a TH1 pattern (e.g.., IL-2 for sustaining and magnifying the expansion of the antigen-specific clones), to a TH2 pattern for the expansion and maturation of B cells, which produce specific antibodies against the "nonself" antigen. Specific antibodies are generally detectable in serum about 15 days from the initial exposure. Re-exposure to the antigen triggers the memory cells, which lead to the appearance of specific antibodies in less than a week. Vaccine development, including vaccine development for cholera intervention, is based on this property of clinical immune surveillance. The timeline of a TH1 to TH2 switch during vCh infection has not been fully characterized to date, but it essential in formulating efficient immunotherapy modalities to challenge and sustain clinical immune surveillance events in cholera patients.

Additional cytokine profiles and distributions of specialized T cell subpopulations have been characterized, which proffer a more complete understanding of the regulatory mechanisms in clinical immune surveillance. For example, a branch of the TH1 pattern of cytokine response leads to a TH17 profile, which sustains chronic inflammatory-like responses, and, new evidence suggests, gut-associated immune activation, such as those induced in irritable bowel syndrome, colitis and the like. Because most of the vCh immune response seems to be gut-associated, the relevance of TH17 to cholera immunity seems unquestionable. Yet, to this date, very little evidence, if any, has been obtained on that secific question.

Last, but not least, a key subpopulation of CD4+ cells, and to some extent, their CD8+ counterparts, is known to be activated (CD25+) and to express FoxP3. These CD4+CD25+FoxP3+ regulatory T cells (Tregs) - although there exist non-activated CD4+/CD8+CD25-FoxP3+ Tregs as well - dampen TH1- mediated T cell activation and proliferation. Tregs are endowed with the functional characteristics, of shutting off antigen-specific clinical immune surveillance to infections by bacteria, such as vCh. But, the role of Tregs in clinical immune surveillance in cholera remains unclear.

Currently, the consensus of the best available research evidence on the immunity to vCh indicates that:

Peripheral blood mononuclear cells, obtained from cholera patients seropositive for vCh-cidal antibodies, and stimulated in vitro with vCh01 show a significant increase in the expression of the marker of T cell activation, CD25, and the cellular marker b7 on T (CD3+) and B (CD19+) cells, which directs T and B lymphocyte homing/migration to gut-associated immune surveillance, as well as increased production of g-interferon, a TH1 cytokine, and IL-13, a TH2 cytokine. The production of IL- 13 is more vigorous at the acute stage of disease, compared to convalescence. In brief, the concerted coordinated systemic events that characterize clinical immune surveillance events in bacterial infections also occur following vCh infection. In cholera, these processes seem targeted to the gut-associated immune compartment early on during the acute phase of the disease [5].

In both children and adults, ingestion of vCh-infected water or food rapidly leads to bacterial colonization of the small intestine, vCh attachment facilitated by its own toxin co-regulated pilus, and a wide range of severity of symptoms, from asymptomatic to mild-to-moderate or severe. Cholera toxin leads intestinal epithelial cells to release chloride, sodium and water via the activated of adenylate cyclase pathway and rise in intracellular cyclic AMP. Patients of all ages show a vigorous 3-step immune surveillance response: a) Lipopolysaccharide - T-cellindependent processing of the vCh serogroup (e.g., O1, O139) (i.e., innate immunity); b) T-cell-dependent response (antigendependent immunity; and c) B antibody response [6].

In addition to a significant rise in pro-inflammatory cytokines (i.e., IL-1b, TNF-a), in bactericidal proteins, and in the migratory potential of neutrophils to the gut lamina propria during acute cholera, these concerted innate immunity events lead to antigenspecific adaptive immune responses. Certain vCh-specific events remain understudied to this date. Case in point, because vCh, typically a non-invasive pathogen, seems to mediate primarily a gut-associated immune activation in loco, which results in the observed rise in intestinal secretory immunoglobulin A (sIgA). Circulating vCh antigen-specific lymphocytes that express guthoming chemokine-receptors peak about 8-10 days following exposure to vCh, but later become undetectable in peripheral blood mononuclear cells, as they have homed to the intestinal mucosa, in part via their b7 membrane marker, where they direct the rise in sIgA. Gut-associated and systemic immunity continue to be intertwined during the cholera episode: vCh-specfic serum antibodies peak 1-3 weeks following infection. Anti-vCh antierum and sIgA titers are well correlated, jointly provide protection against vCh infection at their peak, and decrease to baseline in concert within a year of the original infection. Subsequent exposures trigger immune memory responses, with peaks in both titers in 3-5 days. Memory B cell responses to vCh are detectable for at least 1 year following the original exposure. Cholera vaccines take advantage of this timeline [7]. For example:

As of 2016, the lyophilized CVD 103-HgR (Vaxchora, PaxVax, Redwood City, California), single-dose, live attenuated oral cholera vaccine, was the only approved vaccine intervention by the FDA, and licensed for use in the US, for the prevention of cholera caused by vCh-O1 in adults traveling to cholera-affected areas. Serogroup vCh-O1 is responsible for over 90% of 2.9 million global cases of this water-borne disease and 95,000 deaths, and serogroup O139 is largely responsible for the remaining cases. The body of evidence, which included studies with the currently available lyophilized CVD 103-HgR formulation and studies with oral toxigenic vCh-O1 challenge, consistently indicated high vaccine efficacy and was judged to be GRADE evidence type 1 (evidence from randomized controlled trials or overwhelming evidence from observational studies, which is considered the strongest type of evidence [8, 9, 10]. For safety outcomes, CVD 103-HgR GRADE evidence was limited: type 3 - evidence from observational studies or randomized controlled trials with notable limitations. Nonetheless, CVD 103- HgR is still recommended for adult travelers 18 years of age and above. Most (83%) vaccine recipients show vCh antibody seroconversion within 10 days. The biological reasons why one of five vaccine recipients does not seroconvert remain unknown. The duration of protection conferred by the primary dose beyond the initial 3-month period is unknown, and there are no recommendations for booster doses at this time [11].

Viable alternatives to live attenuated oral cholera vaccines are the whole-cell killed oral cholera (wc-kOC) vaccines. A 2017 multidimensional systematic review, including randomized clinical trials (n=7) and observational cohort studies (n=6) from English, Spanish, French, and Chinese peer-reviewed literature and obtained from the national Library of Medicine (PubMed, Embase, Scopus, the Cochrane Review Library, and ISI Web of Science) were used to obtain the consensus of on efficacy and effectiveness of wc-kOC vaccines for protection against cholera among population at risk. This comparative efficacy and effectiveness research and analysis for practice (8-10) study established the consensus for a short-term average (within 2 years protection) two-dose efficacy of 58% (CI95: 42-69, I2=58%) and effectiveness of 76% (CI95: 62-85, I2=0). Nonetheless, the average two-dose efficacy in children younger than 5 years was significantly lower (median: 30% vs. 64%, p<0.05), compared to children 5 years or older. Moreover, the two-dose efficacy estimates of wc-kOC vaccines were similar during the longer term first 2-year period following vaccination, with estimates of 56% (median 56% first year CI95: 42-66, I2=45%; second year 59% CI95: 49-67, I2=0; p>0.05), but significantly decreased (p<0.05) in subsequent years (39% CI95: 13 to 57, I2=48% third year; 26% CI95: -46 to 63, I2=74% fourth year) [12]. Among these, WHO recommends a few prequalified wc-kOCV's:

Dukoral® (Crucell, Netherlands, 1990), denatured vCh-) 1 antigen, supplemented with the classical and El Tor biotypes the Ogawa and Inaba serotypes: efficacy peak in children 2 years of age (~85%) with a moderately steep decline (~50%) from 5 years of age onward. Dukoral® is recommended for those traveling from non-endemic to endemic regions.

Shanchol™ (Shantha, Biotechnics-Sanofi Pasteur, India), a bivalent (o1 and O139) wc-kOCV that lacks the toxigenic B subunit: two-dose efficacy of Shanchol™ is approximately 67% among both children and adults.

Euvichol™ (EuBiologics Co., Ltd., South Korea), and similarly mORC-Vax™ (VaBiotech, Vietnam), although the latter has not t yet received WHO prequalification. Both vaccines were effective in containing Vietnam's cholera-endemic 1997-2013 outbreak.

## Conclusion

Cholera is a prototypical non-inflammatory infection, which causes no flamboyant inflammation to the intestinal mucosa or the architectural integrity of the small bowel. It is preventable by ensuring access to safe water and sanitation, remains endemic in over 50 countries to this day. Its etiological factor, vCh, is responsible for epidemics that are aggressive both in morbidity and mortality. Since 1817, seven cholera pandemics have spread across the world, although early descriptions of cholera can be traced to the 5th century BC. The 7th pandemic of modern times began in 1961 and affected at least 3-5 million people year after year. It is debated whether the current cholera epidemic in Yemen and potentially spreading across the Middle East in fact represents the onset of the 8th cholera pandemic. Outbreaks engage a rapid and vigorous response from global health task forces, which to date primarily utilizes vaccination modalities, to the detriment of alternate intervention routes directed to innate and antigen-dependent cell-mediated immune events.

Cholera is a gastro-intestinal illness, which presents with dramatic diarrheal purging. Patient management requires controlling dehydration by means of aggressive fluid replacement as well as mico-nutrient replenishment. Effective therapy can decrease mortality from over 50% to less than 0.2%, when appropriate antibiotics are administered.

The available vaccines are good modalities to slow the progress of the disease across a population at risk by protecting patients at risk from infection with vCh. Oral vaccination protocols are tailored against vCh-O1 primarily and secondarily vCh- O139. Vaccines based on live vCh strains may also cause significantly more risk than whole cell-killed vaccines (wc-kOC). The best evidence base demonstrates that wc-kOCV's are safe, efficacious (i.e., immunogenic), cost-effective and simple to administer [13, 14, 15, 16].

Vaccine modalities alone do little, however, to prevent the physiopathology that afflicts individual patients. In addition, little is done presently from the perspective of clinical immunology for patients at the early stages of vCh infection.
